# Neuronal Igfbp2 deficiency in the prefrontal cortex impairs cognition through synaptic dysfunction in male mice

**DOI:** 10.1016/j.isci.2026.115629

**Published:** 2026-04-06

**Authors:** Weiming Zhao, Yanan Gao, Yichan Wang, Ke Peng, Shaoyong Song, Yufan Yang, Baojian Zhao, Xisheng Shan, Li Deng, Ruixia Weng, Hong Liu, Xiaowen Meng, Huayue Liu, Fuhai Ji

**Affiliations:** 1 Department of Anesthesiology, First Affiliated Hospital of Soochow University, Suzhou, Jiangsu, China; 2 Institute of Anesthesiology, Soochow University, Suzhou, Jiangsu, China; 3 Ambulatory Surgery Center, First Affiliated Hospital of Soochow University, Suzhou, Jiangsu, China; 4 Department of Anesthesiology, Nanjing Stomatological Hospital, Affiliated Hospital of Medical School, Nanjing University, Nanjing, Jiangsu, China; 5 Department of Anesthesiology and Pain Medicine, University of California Davis Health System, Sacramento, CA 95817, USA; 6 Department of Gastroenterology, First Affiliated Hospital of Soochow University, Suzhou, Jiangsu, China

**Keywords:** biochemistry, neuroscience, Cell biology

## Abstract

Insulin-like growth factor binding protein 2 (Igfbp2) is known to participate in brain development and synaptic regulation, but its specific contribution to prefrontal cortex (PFC)-dependent cognitive function remains unclear. Here, we demonstrate that neuronal Igfbp2 in the PFC plays a critical role in maintaining cognitive performance and synaptic integrity. Neuron-specific Igfbp2 knockdown impaired recognition and spatial memory without altering emotional or locomotor behaviors. Mechanistically, loss of Igfbp2 reduced dendritic spine density, downregulated synapsin-1 and PSD-95 expression, and weakened excitatory synaptic transmission. Remarkably, the local infusion of recombinant Igfbp2 protein restored synaptic architecture and rescued cognitive deficits. Together, these findings identify neuronal Igfbp2 as an essential regulator of PFC-dependent cognition and highlight its potential as a therapeutic target for cognitive dysfunction.

## Highlights

• Neuron-specific Igfbp2 knockdown in PFC impairs cognition in adult male mice
• Loss of Igfbp2 disrupts synaptic integrity and neuronal excitability in PFC
• Exogenous supplementation with Igfbp2 in PFC rescues synaptic and cognitive deficits

## Graphical abstract

**Figure:**
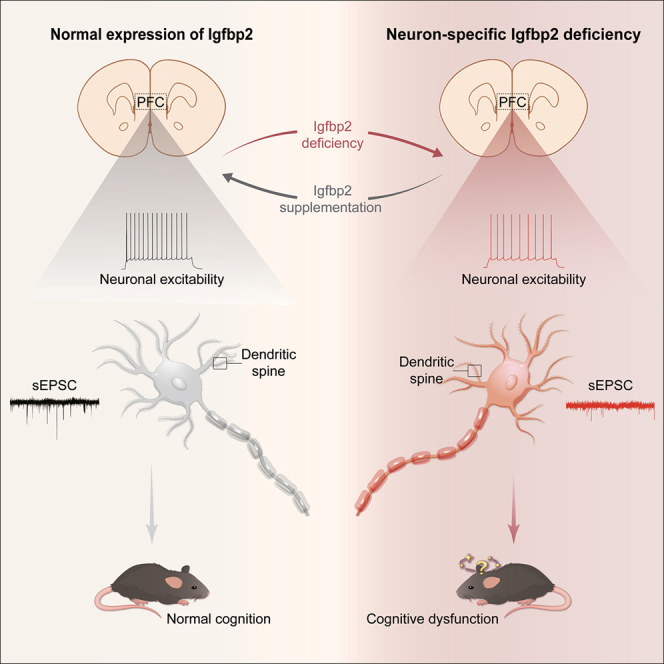

## Introduction

The insulin-like growth factor binding protein (Igfbp) family regulates cell growth, differentiation, and metabolism by modulating the bioavailability and activity of insulin-like growth factors (IGFs).^1^^,^^2^^,^^3^ Among these proteins, Igfbp2 is the most abundant in cerebrospinal fluid and exhibits high expression levels in the central nervous system throughout development, from the embryonic period into adulthood.^4^ Igfbp2 is primarily synthesized by neurons and astrocytes, functioning through autocrine and/or paracrine pathways to regulate neuronal excitability, synaptic transmission, as well as central nervous system injury and repair.^5^ Recent evidence indicates that Igfbp2 in hippocampal neurons is critical for early cognitive development through the regulation of neuronal plasticity.^6^ Our previous study demonstrated that Igfbp2 in glutamatergic neurons within paraventricular thalamus afferents to the central amygdala mediates neonatal anesthesia-induced fear memory deficits.^7^ In addition, Igfbp2 exerts neuroprotective effects by reducing Aβ-induced tau phosphorylation and neuronal death in Alzheimer’s disease.^8^ Another study also demonstrated that the exogenous supplementation of recombinant Igfbp2 protein can alleviate posttraumatic stress disorder by facilitating brain plasticity.^9^ Collectively, these findings underscore the broad and pivotal role of Igfbp2 in regulating cognitive processes across multiple brain regions and pathological conditions.

The prefrontal cortex (PFC) plays a pivotal role in cognition and higher-level executive functions. Accumulating evidence indicates that neural network disturbances within the PFC are major contributors to cognitive dysfunction.^10^^,^^11^ Aging-related structural and functional changes in the PFC, including reduced connectivity and volumetric loss, frequently precede the broader cognitive impairments observed in dementia syndromes.^12^ PFC dysfunction is also a prominent feature of neurodegenerative diseases such as Alzheimer’s disease, where it contributes to early cognitive decline characterized by deficits in executive function and memory retrieval.^13^ However, whether Igfbp2 in the PFC directly regulates cognitive function remains largely unknown.

In this study, we investigated the role of neuronal Igfbp2 in the PFC in mediating cognitive function. Using adeno-associated virus (AAV)-mediated gene knockdown, we found that the neuron-specific depletion of Igfbp2 in the PFC resulted in cognitive dysfunction in mice. Mechanistically, Igfbp2 knockdown decreased dendritic spine density and impaired excitatory synaptic transmission within the PFC. Importantly, local supplementation with exogenous recombinant Igfbp2 protein rescued the cognitive deficits induced by Igfbp2 knockdown through the restoration of dendritic spine density and excitatory synaptic transmission. Therefore, our results reveal that Igfbp2 in PFC neurons is essential for cognitive function and highlight its potential as a therapeutic target for cognitive impairment.

## Results

### Igfbp2 is predominantly expressed in PFC neurons

**Figure 1:**
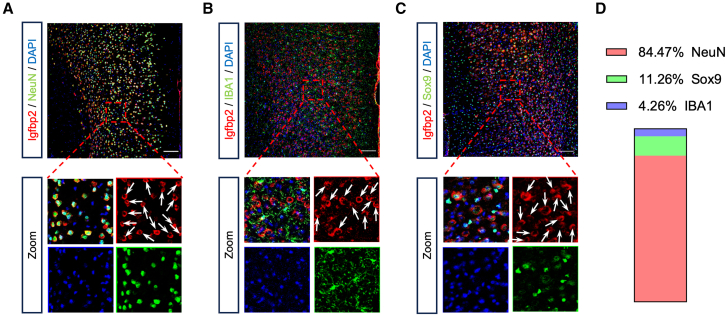
Igfbp2 is predominantly expressed in the PFC neurons (A) Representative immunofluorescence image of Igfbp2 (red) and NeuN (green) in the PFC. (B) Representative immunofluorescence image of Igfbp2 (red) and IBA1 (green) in the PFC. (C) Representative immunofluorescence image of Igfbp2 (red) and Sox9 (green) in the PFC. (D) Quantitative analysis of Igfbp2 colocalization with different cell types in the PFC. Scale bars, 100 μm; *n* = 4 brain sections from 4 mice per group.

Although Igfbp2 in hippocampal pyramidal neurons has been shown to be essential for early cognitive development,^6^ its role in cognitive regulation within the PFC remains poorly understood. To address this question, we first examined the cellular distribution of Igfbp2 in the PFC. Brain tissue sections were prepared and subjected to double immunofluorescence staining for Igfbp2 (Santa Cruz, sc-515134) in combination with NeuN (neuronal marker), Sox9 (astrocytic marker), or IBA1 (microglial marker). Our results showed that Igfbp2 was predominantly colocalized with NeuN, while exhibiting sparse colocalization with Sox9 or IBA1 in the PFC (Figures 1A–1D). To further validate antibody specificity and assess staining reproducibility, we performed co-immunostaining using a second independent anti-Igfbp2 antibody (R&D Systems, AB5535) together with the same neuronal, astrocytic, and microglial markers. Consistent with our initial observation, the Igfbp2 signal was predominantly detected in NeuN-positive neurons, with minimal colocalization with Sox9 or IBA1 (Figure S1). Together, the concordant results obtained using two independent Igfbp2 antibodies indicate that Igfbp2 is primarily localized to neurons in the PFC. These findings indicate that Igfbp2 is primarily localized to neurons in the PFC, providing a cellular basis for investigating its potential role in cognitive function.

### Neuron-specific Igfbp2 deficiency in the PFC induces cognitive dysfunction

**Figure 2:**
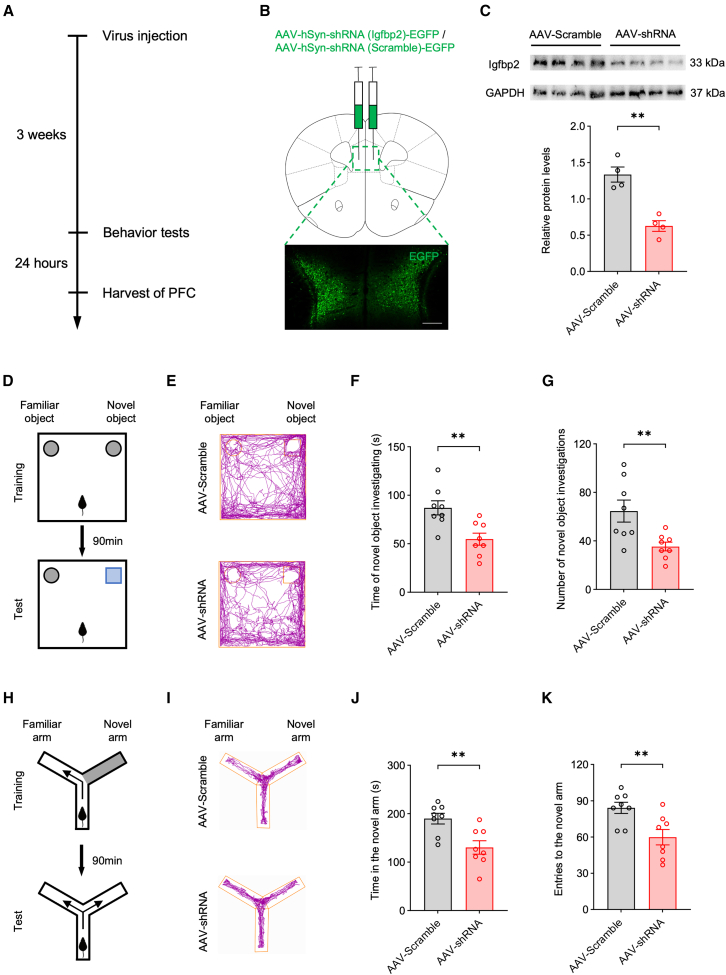
Neuron-specific Igfbp2 deficiency in the PFC induces cognitive dysfunction (A) Schematic of the experimental design. (B) Diagram of viral injection into the PFC (top) and representative image of viral expression (bottom); scale bars, 500 μm. (C) Representative western blot bands (top) and quantification of Igfbp2 protein levels in the PFC (bottom); *n* = 4 mice per group. (D) Schematic of the novel object recognition test, depicting the training phase (top) and the test phase (bottom). (E) Representative trajectory plots from the novel object recognition test. (F) Quantification of time spent investigating the novel object during the test phase; *n* = 8 mice per group. (G) Quantification of the number of investigations of the novel object during the test phase; *n* = 8 mice per group. (H) Schematic of the Y-maze test, depicting the training phase (top) and the test phase (bottom). (I) Representative trajectory plots from the Y-maze test. (J) Quantification of time spent in the novel arm during the test phase; *n* = 8 mice per group. (K) Quantification of the number of entries into the novel arm during the test phase; *n* = 8 mice per group. Analyzed by unpaired *t* test; ∗∗*p* < 0.01. Data are presented as means ± SEM.

To examine the functional consequences of Igfbp2 deficiency in PFC neurons, we achieved neuron-specific Igfbp2 knockdown by bilaterally injecting AAV-hSyn-shRNA (Igfbp2)-EGFP (AAV-shRNA) into the PFC of wild-type mice, with AAV-hSyn-shRNA (Scramble)-EGFP (AAV-Scramble) serving as a control. Mice then underwent a comprehensive battery of behavioral assays (Figure 2A). All behavioral experiments were performed in adult mice. At 3 weeks post-injection, PFC tissue was collected 24 h after the completion of the behavioral tests to assess knockdown efficiency. Viral infection in the PFC was confirmed by EGFP fluorescence (Figure 2B), and Igfbp2 knockdown efficiency was quantified at the protein level by western blot analysis (Figure 2C). In addition, Igfbp2 knockdown was further validated at the mRNA level by quantitative RT-PCR and at the cellular level by Igfbp2 immunofluorescence co-staining with viral EGFP (Figure S2).

In the novel object recognition test, the AAV-shRNA group spent significantly less time investigating and made fewer approaches to the novel object compared with the AAV-Scramble group (Figures 2D–2G). Similarly, in the Y-maze test, the AAV-shRNA group exhibited significantly reduced time in and fewer entries into the novel arm compared to the AAV-Scramble group (Figures 2H–2K). These results indicated that neuron-specific knockdown of Igfbp2 in the PFC impairs cognitive performance.

In contrast, both the forced swim test and tail suspension test revealed no significant differences in immobility time between the AAV-shRNA and AAV-Scramble groups (Figure S3), suggesting that Igfbp2 deficiency in PFC neurons did not induce depression-like behavior. Similarly, the elevated plus maze test revealed no significant differences in open-arm time between groups, and the open field test showed comparable time spent in the central zone as well as total distance traveled (Figure S4), indicating that neuron-specific Igfbp2 knockdown in the PFC did not affect anxiety-like behavior or locomotor activity. In addition, body weight remained unaffected by Igfbp2 deficiency in PFC (Figure S5).

Collectively, these findings demonstrate that Igfbp2 knockdown in PFC neurons selectively impairs cognitive function without affecting mood-related behaviors, locomotor activity, or body weight.

### Neuron-specific Igfbp2 deficiency in the PFC leads to synaptic dysfunction

**Figure 3:**
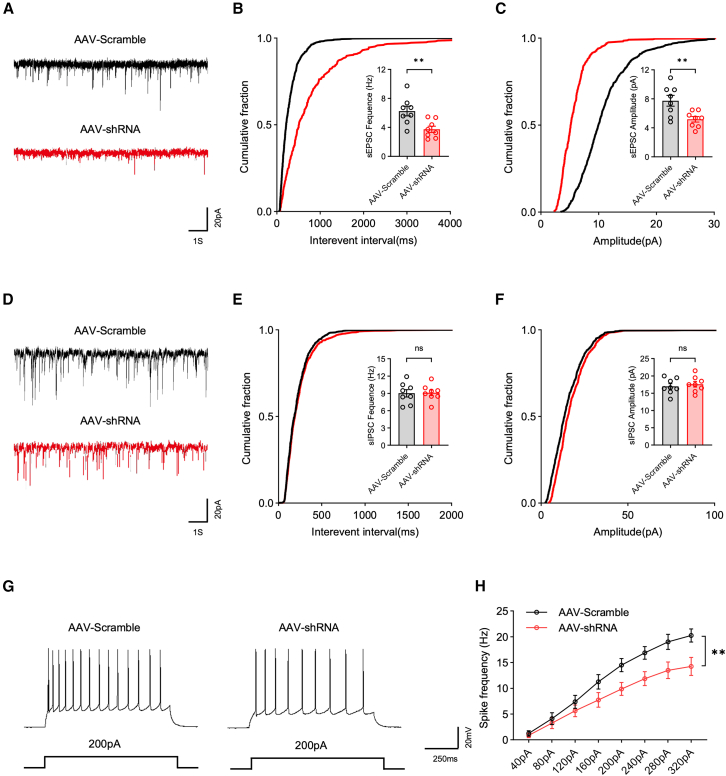
Neuron-specific Igfbp2 deficiency in the PFC reduces excitatory synaptic transmission and neuronal excitability (A) Representative traces of sEPSC recorded from PFC neurons in AAV-scramble (top) and AAV-shRNA (bottom) groups. (B) Cumulative probability plots (main panel) and quantification (insert panel) of sEPSC frequency in AAV-Scramble and AAV-shRNA groups. (C) Cumulative probability plots (main panel) and quantification (insert panel) of sEPSC amplitude in AAV-Scramble and AAV-shRNA groups. (D) Representative traces of sIPSC recorded from PFC neurons in AAV-Scramble (top) and AAV-shRNA (bottom) groups. (E) Cumulative probability plots (main panel) and quantification (insert panel) of sIPSC frequency in AAV-Scramble and AAV-shRNA groups. (F) Cumulative probability plots (main panel) and quantification (insert panel) of sIPSC amplitude in AAV-Scramble and AAV-shRNA groups. (G) Representative traces of AP elicited by 200 pA current injections in PFC neurons from AAV-Scramble (left) and AAV-shRNA (right) groups. (H) Quantification of AP firing frequency in response to a range of current injections in PFC neurons from AAV-Scramble and AAV-shRNA groups. Analyzed by unpaired *t* test in B, C, E, and F, and two-way repeated-measure ANOVA in H; *n* = 8 neurons from 4 mice per group; ns: not significant, ∗∗*p* < 0.01. Data are presented as means ± SEM.

Synaptic dysfunction in the PFC is strongly implicated in cognitive decline,^14^^,^^15^ Previous studies have demonstrated that Igfbp2 can modulate synaptic function by regulating synaptic connectivity and dendritic spine density.^16^^,^^17^ Therefore, we investigated whether Igfbp2 deficiency in PFC neurons alters synaptic transmission properties. Whole-cell patch-clamp recordings were performed on virus-infected PFC neurons to assess synaptic function. We found that both the frequency and amplitude of spontaneous excitatory postsynaptic currents (sEPSCs) were significantly reduced in the AAV-shRNA group compared with the AAV-Scramble group (Figures 3A–3C), indicating that Igfbp2 deficiency in PFC neurons impairs excitatory synaptic transmission. In contrast, no significant differences were detected in the frequency or amplitude of spontaneous inhibitory postsynaptic currents (sIPSCs) between the two groups (Figures 3D–3F). Furthermore, PFC neurons in the AAV-shRNA group exhibited markedly reduced intrinsic excitability compared with those in the AAV-Scramble group, as evidenced by decreased action potential (AP) firing frequency in response to depolarizing current injections (Figures 3G and 3H). Importantly, the overall neuronal density in the PFC remained unchanged following neuron-specific Igfbp2 knockdown (Figure S6), ruling out neuronal loss as a confounding factor.

**Figure 4:**
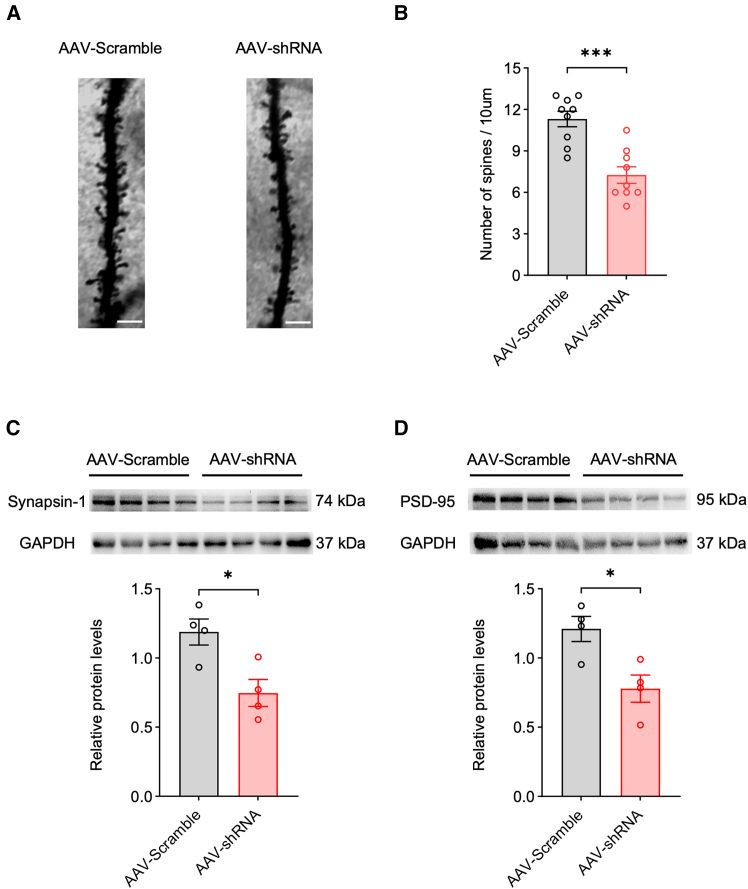
Neuron-specific Igfbp2 deficiency in the PFC disrupts synaptic integrity (A) Representative images of dendritic spines in the PFC from AAV-Scramble (left) and AAV-shRNA (right) groups; scale bars, 10 μm. (B) Quantification of dendritic spine density (number per 10 μm) in the PFC from AAV-Scramble and AAV-shRNA groups; *n* = 9 brain sections from 3 mice per group. (C) Representative western blot bands (top) and quantification of synapsin-1 protein levels (bottom) in the PFC from AAV-scramble and AAV-shRNA groups; *n* = 4 mice per group. (D) Representative western blot bands (top) and quantification of PSD-95 protein levels (bottom) in the PFC from AAV-Scramble and AAV-shRNA groups; *n* = 4 mice per group. Analyzed by unpaired *t* test; ∗*p* < 0.05 and ∗∗∗*p* < 0.001. Data are presented as means ± SEM.

To further investigate the structural correlates of impaired synaptic transmission, we assessed dendritic spine densities in the PFC using Golgi-Cox staining. The AAV-shRNA group showed a significantly lower dendritic spine density compared with the AAV-Scramble group (Figures 4A and 4B), suggesting that reduced spine density may contribute to the observed alterations in synaptic transmission. Given that synapsin-1 (a presynaptic marker) and PSD-95 (a postsynaptic marker) are key regulators of synaptic plasticity through their respective roles in neurotransmitter release and glutamate receptor trafficking,^18^^,^^19^^,^^20^ we next examined their expression levels. Consistent with the reduction in spine density, western blot analysis revealed that the protein expression levels of both synapsin-1 and PSD-95 were significantly decreased in the AAV-shRNA group compared with the AAV-Scramble group (Figures 4C and 4D).

Taken together, these findings indicate that Igfbp2 knockdown in PFC neurons contributes to synaptic dysfunction characterized by impairing excitatory synaptic transmission, reducing neuronal excitability, and decreasing synaptic structural integrity, which may underlie the cognitive deficits observed following Igfbp2 depletion.

### Exogenous supplementation of Igfbp2 in the PFC rescues cognitive deficits induced by neuron-specific Igfbp2 deficiency

**Figure 5:**
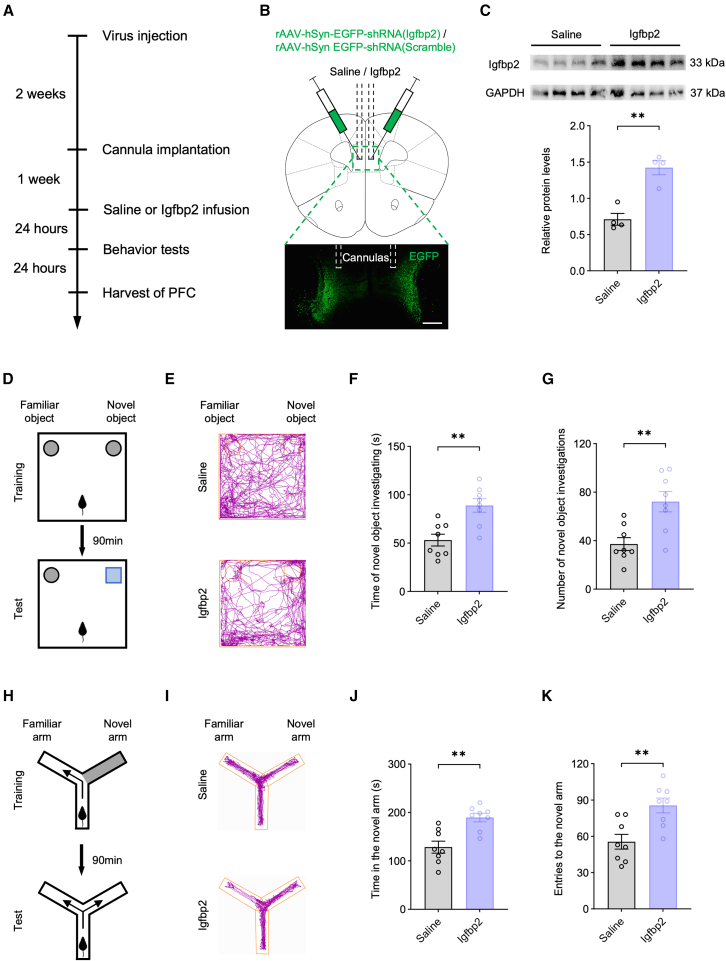
Exogenous supplementation of Igfbp2 in the PFC rescues cognitive deficits induced by neuron-specific Igfbp2 deficiency (A) Schematic of the experimental design. (B) Diagram shows virus injection and cannula placement into the PFC (top), and a representative image of cannula position and viral expression in the PFC (bottom); scale bars, 500 μm. (C) Representative western blot bands (top) and quantification of Igfbp2 protein levels in the PFC (bottom); *n* = 4 mice per group. (D) Schematic of the novel object recognition test, depicting the training phase (top) and the test phase (bottom). (E) Representative trajectory plots from the novel object recognition test. (F) Quantification of time spent investigating the novel object during the test phase; *n* = 8 mice per group. (G) Quantification of the number of investigations of the novel object during the test phase; *n* = 8 mice per group. (H) Schematic of the Y-maze test, depicting the training phase (top) and the test phase (bottom). (I) Representative trajectory plots from the Y-maze test. (J) Quantification of time spent in the novel arm during the test phase; *n* = 8 mice per group. (K) Quantification of the number of entries into the novel arm during the test phase; *n* = 8 mice per group. Analyzed by unpaired *t* test; ∗∗*p* < 0.01. Data are presented as means ± SEM.

Building upon these findings, we next investigated whether exogenous Igfbp2 supplementation in the PFC could ameliorate cognitive deficits induced by neuron-specific Igfbp2 deficiency. Two weeks after viral injection, we stereotaxically implanted bilateral guide cannulas into the PFC. One week later, recombinant Igfbp2 protein or vehicle (saline) was locally infused 24 h prior to behavioral testing (Figure 5A). Representative coronal brain sections confirmed successful viral transduction and accurate cannula placement within the PFC (Figure 5B). Western blot analysis further demonstrated that Igfbp2 infusion significantly increased Igfbp2 protein levels in the PFC compared with saline infusion (Figure 5C).

Behavioral analysis showed that the Igfbp2-treated group spent significantly more time exploring and made more approaches to the novel object compared with the saline-treated group in the novel object recognition test (Figures 5D–5G). Similarly, in the Y-maze test, Igfbp2-treated mice exhibited significantly increased time in and more entries into the novel arm relative to the saline group (Figures 5H–5K). By contrast, mice infused with heat-denatured Igfbp2 did not show increases in exploration time or approaches to the novel object in the novel object recognition test, nor increases in time spent in or entries into the novel arm in the Y-maze test, compared with saline-treated mice (Figure S7). These findings indicate that exogenous Igfbp2 supplementation in the PFC effectively rescues cognitive dysfunction induced by neuron-specific Igfbp2 knockdown.

### Exogenous supplementation of Igfbp2 in the PFC restores synaptic function impaired by neuron-specific Igfbp2 deficiency

**Figure 6:**
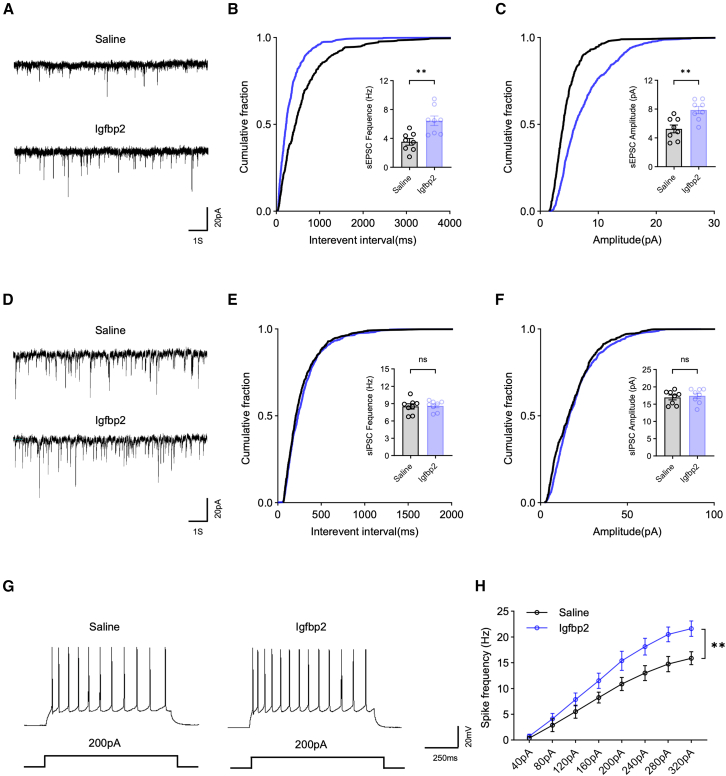
Exogenous supplementation of Igfbp2 in the PFC restores excitatory synaptic transmission and neuronal excitability impaired by neuron-specific Igfbp2 deficiency (A) Representative traces of sEPSC recorded from PFC neurons in saline (top) and Igfbp2 (bottom) groups. (B) Cumulative probability plots (main panel) and quantification (insert panel) of sEPSC frequency in saline (top) and Igfbp2 (bottom) groups. (C) Cumulative probability plots (main panel) and quantification (insert panel) of sEPSC amplitude in saline and Igfbp2 groups. (D) Representative traces of sIPSC recorded from PFC neurons in saline (top) and Igfbp2 (bottom) groups. (E) Cumulative probability plots (main panel) and quantification (insert panel) of sIPSC frequency in saline and Igfbp2 groups. (F) Cumulative probability plots (main panel) and quantification (insert panel) of sIPSC amplitude in saline and Igfbp2 groups. (G) Representative traces of AP elicited by 200 pA current injections in PFC neurons from saline (left) and Igfbp2 (right) groups. (H) Quantification of AP firing frequency in response to a range of current injections in PFC neurons from Saline (left) and Igfbp2 (right) groups. Analyzed by unpaired *t* test in B, C, E, and F, and two-way repeated-measure ANOVA in H; *n* = 8 neurons from 4 mice per group; ns: not significant, ∗∗*p* < 0.01. Data are presented as means ± SEM.

Given that Igfbp2 deficiency impaired synaptic function, we next examined whether exogenous Igfbp2 supplementation could reverse these deficits. Whole-cell patch-clamp recordings revealed that the Igfbp2-treated group exhibited significantly increased frequency and amplitude of sEPSC compared with the saline-treated group (Figures 6A–6C), indicating the restoration of excitatory synaptic transmission. No significant differences were observed in either the frequency or amplitude of sIPSC between the two groups (Figures 6D–6F). Furthermore, Igfbp2 supplementation markedly increased AP firing frequency in PFC neurons compared with saline treatment (Figures 6G and 6H), demonstrating recovery of neuronal excitability.

**Figure 7:**
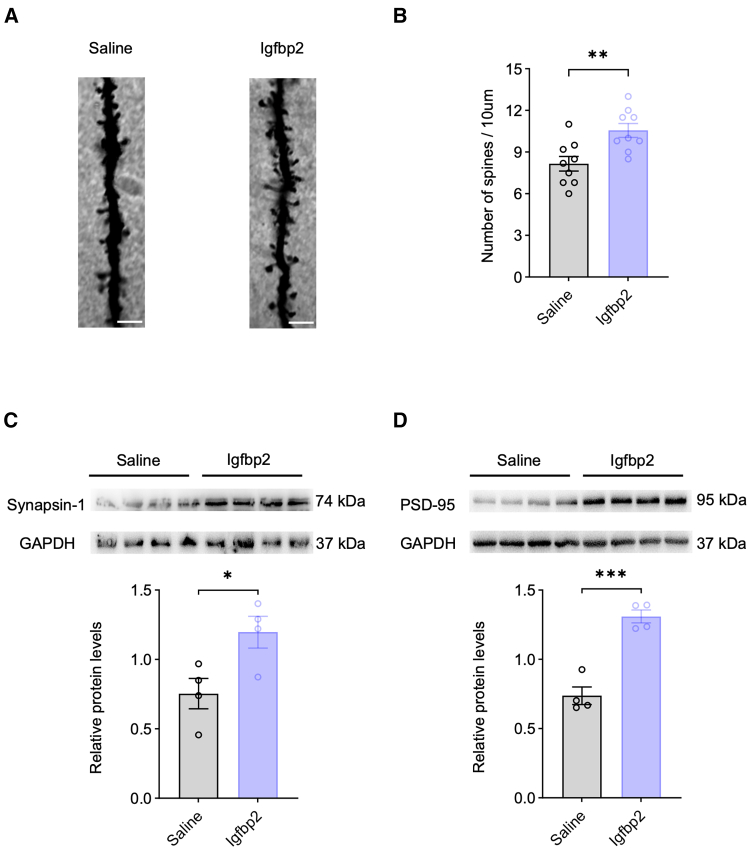
Exogenous supplementation of Igfbp2 in the PFC restores synaptic integrity disrupted by neuron-specific Igfbp2 deficiency (A) Representative images of dendritic spines in the PFC from saline (left) and Igfbp2 (right) groups; scale bars, 10 μm. (B) Quantification of dendritic spine density (number per 10 μm) in the PFC from saline and Igfbp2 groups; *n* = 9 brain sections from 3 mice per group. (C) Representative western blot bands (top) and quantification of synapsin-1 protein levels (bottom) in the PFC from AAV-Scramble and AAV-shRNA groups; *n* = 4 mice per group. (D) Representative western blot bands (top) and quantification of PSD-95 protein levels (bottom) in the PFC from AAV-Scramble and AAV-shRNA groups; *n* = 4 mice per group. Analyzed by unpaired *t* test; ∗*p* < 0.05 and ∗∗∗*p* < 0.001. Data are presented as means ± SEM.

Morphological and molecular analyses further supported these electrophysiological findings. Golgi-Cox staining revealed that the Igfbp2-treated group showed a significantly higher dendritic spine density compared with the saline group (Figures 7A and 7B). Consistently, western blot analysis showed that protein expression levels of both synapsin-1 and PSD-95 were significantly increased in the Igfbp2 group relative to the saline group (Figures 7C and 7D).

Collectively, these results demonstrate that exogenous Igfbp2 supplementation effectively restores synaptic structure and function impaired by Igfbp2 knockdown in PFC neurons, providing a mechanistic basis for the observed cognitive rescue.

## Discussion

**Figure 8:**
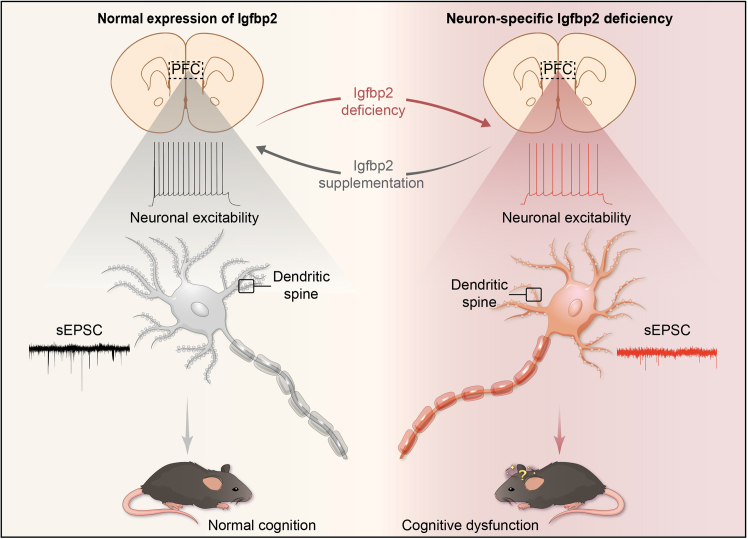
Schematic summary of this study Neuron-specific Igfbp2 deficiency in the PFC impairs cognitive function by disrupting synaptic integrity, excitatory transmission, and neuronal activity, whereas exogenous Igfbp2 supplementation mitigates cognitive deficits by restoring synaptic integrity, excitatory transmission, and neuronal activity. PFC prefrontal cortex, sEPSC spontaneous excitatory postsynaptic currents, Igfbp2 insulin-like growth factor-binding protein 2.

Igfbp2 has been shown to play important roles during brain development, including the regulation of neuronal growth and synaptic formation. However, its contribution to cognitive function has remained incompletely understood. In this study, we identified a previously unrecognized role for Igfbp2 in PFC neurons as a critical regulator of cognition (Figure 8). Neuron-specific deficiency of Igfbp2 led to significant cognitive deficits in male mice, accompanied by impaired excitatory synaptic transmission and compromised synaptic structural integrity, which collectively disrupted synaptic function. Importantly, local supplementation with recombinant Igfbp2 protein in the PFC reversed both synaptic and cognitive deficits, establishing Igfbp2 as an essential factor for sustaining PFC-dependent cognitive processes.

Our study demonstrates that Igfbp2 is predominantly expressed in neurons within the PFC, with minimal expression in astrocytes or microglia. To determine whether neuronal Igfbp2 in the PFC plays a critical role in cognitive function, we employed an AAV vector expressing Igfbp2 short hairpin RNAs under the neuron-specific hSyn promoter to achieve targeted Igfbp2 knockdown, followed by comprehensive behavioral characterization. We validated Igfbp2 knockdown efficiency by collecting PFC tissue 24 h after the final behavioral assay, three weeks after AAV injection, and confirmed a significant reduction in Igfbp2 expression by quantitative RT-PCR, western blot analysis, and immunofluorescence. Although we did not assess this longitudinally beyond our testing window, a prior intracranial rAAV study reported that expression is detectable within approximately 1 week, becomes robust by approximately 3 weeks post-injection, and remains relatively stable thereafter, supporting stable knockdown throughout our behavioral testing period.^21^ We then assessed the behavioral consequences of Igfbp2 reduction in the PFC and found that neuron-specific Igfbp2 knockdown selectively impaired cognitive performance, as evidenced by deficits in both novel object recognition and Y-maze tests. Notably, anxiety-like behavior, depression-like behavior, and locomotor activity remained unaffected, indicating that the effects of Igfbp2 deficiency were specific to cognitive domains.

Although the PFC is implicated in both cognitive and affective regulation, Igfbp2 knockdown in our study resulted in a selective cognitive deficit without significantly altering anxiety- or depression-like behaviors. Specifically, Igfbp2 deficiency reduced dendritic spine density, decreased synaptic protein expression, and impaired excitatory synaptic transmission and neuronal excitability in PFC neurons. These Igfbp2-dependent synaptic and intrinsic changes are fundamental for PFC-dependent information processing and are likely rate-limiting for working memory and recognition performance. By contrast, affect-related behaviors often rely on broader, distributed networks and are more context- or state-dependent. Thus, the extent of Igfbp2 reduction achieved here may be sufficient to disrupt cognitive computations under baseline conditions but insufficient to produce measurable changes in baseline anxiety- or depression-like assays. Consistent with this interpretation, our findings align with and extend previous observations. Earlier work demonstrated that Igfbp2 in hippocampal neurons regulates early cognitive development through modulating neuronal plasticity.^6^ More recently, Igfbp2 in basolateral amygdala (BLA) astrocytes was shown to be essential for fear memory expression without affecting locomotor activity or anxiety-like behaviors, consistent with the behavioral specificity observed in our findings.^22^ Furthermore, another study demonstrated that astrocyte-derived Igfbp2 in the amygdala forms a multiday molecular trace that stabilizes fear memory and sustains long-term synaptic plasticity.^23^ Our study builds upon this foundation by providing direct evidence that Igfbp2 expression in PFC neurons is indispensable for adult cognitive function, thereby extending the role of Igfbp2 to higher-order executive processes beyond hippocampal- and amygdala-dependent learning and memory.

Synaptic dysfunction in the PFC is a cardinal feature of cognitive decline in neurodegenerative disorders such as Alzheimer’s disease.^24^^,^^25^^,^^26^ Previous studies have demonstrated that Igfbp2 is critical for maintaining synaptic function through its contributions to synaptic structural organization and neurotransmission regulation.^17^ Our findings revealed that Igfbp2 knockdown in PFC neurons impaired excitatory synaptic transmission and reduced neuronal excitability. In addition, Igfbp2 depletion disrupted synaptic structural integrity, as evidenced by decreased dendritic spine density and reduced expression of key synaptic markers (synapsin-1 and PSD-95) in the PFC. Notably, Igfbp2 knockdown did not alter the overall neuronal density in the PFC, ruling out neuronal loss as a contributing factor to the observed deficits. These findings are consistent with recent studies demonstrating that Igfbp2 enhances neuronal excitability and promotes dendritic spine maturation through autocrine or paracrine signaling mechanisms.^16^^,^^17^^,^^27^ Overall, these results strongly suggest that Igfbp2 in PFC neurons is essential for cognitive function through its regulation of synaptic structure and activity.

Recent evidence has shown that an Igfbp2-derived peptide promotes neuroplasticity and rescues cognitive and behavioral impairments in a mouse model of Phelan-McDermid syndrome.^17^ Consistent with these findings, our results suggest that exogenous Igfbp2 supplementation in the PFC reverses the deficits caused by Igfbp2 knockdown, as evidenced by the restoration of excitatory synaptic transmission, neuronal excitability, dendritic spine density, and synaptic protein expression. As a modulator of IGF signaling, Igfbp2 binds to IGFs to regulate their bioavailability and localization.^4^ Previous studies have shown that Igfbp2 can potentiate IGF2 receptor signaling, leading to the activation of downstream ERK1/2 pathways that promote dendritic spine maturation and upregulate synaptic protein expression in forebrain neurons, including postsynaptic scaffold proteins such as PSD-95 and other synaptic components.^28^ These mechanisms collectively explain how Igfbp2 may regulate these synaptic proteins in PFC neurons.

The restoration of synaptic integrity and excitatory activity by exogenous Igfbp2 suggests that this protein serves dual functions in the nervous. First, Igfbp2 acts as a structural regulator of synaptic architecture by maintaining dendritic spines and stabilizing synaptic proteins. Second, it functions as a functional modulator of neuronal excitability and synaptic transmission. These dual roles are consistent with previous reports showing that Igfbp2 can interact with extracellular matrix components to modulate growth factor bioavailability while simultaneously influencing intrinsic neuronal membrane properties.^29^ Notably, Igfbp2 deficiency in hippocampal pyramidal neurons has been shown to cause cognitive impairments that can be ameliorated by recombinant Igfbp2 supplementation.^6^ Our findings parallel these observations in the hippocampus, demonstrating that Igfbp2 supplementation can similarly rescue cognitive dysfunction resulting from Igfbp2 deficiency in PFC neurons. Collectively, these findings underscore the importance of Igfbp2 across multiple brain regions for maintaining synaptic function and cognitive integrity.

In summary, our study reveals that Igfbp2 in PFC neurons is critical for cognitive function through its dual roles in maintaining synaptic structural integrity and modulating excitatory neurotransmission. Igfbp2 deficiency disrupts these processes, leading to cognitive impairment, whereas exogenous Igfbp2 supplementation effectively restores synaptic function and rescues cognitive deficits. These findings advance our understanding of Igfbp2 function in the brain and suggest that targeting Igfbp2 may hold therapeutic potential for treating cognitive disorders associated with synaptic dysfunction.

### Limitations of the study

Despite these strengths, several limitations warrant consideration. First, our study exclusively examined male mice, which may constrain the generalizability of our findings. Therefore, future studies including both sexes will be necessary to determine whether these effects extend to females and to directly compare males and females. Second, while recombinant Igfbp2 protein effectively rescued behavioral and synaptic deficits, the precise downstream molecular pathways mediating these effects remain incompletely defined. Future studies employing cell-type-specific transcriptomic and proteomic approaches will be necessary to elucidate the signaling cascades through which Igfbp2 regulates synaptic function and cognition. Third, the long-term consequences of Igfbp2 modulation on cognitive performance and synaptic plasticity are unknown. Given the dynamic regulation of Igfbp2 expression by developmental stage, neuronal activity, and environmental factors, it will be important to determine whether transient Igfbp2 supplementation yields sustained cognitive benefits or whether continuous treatment is required. Finally, although the exogenous supplementation of Igfbp2 significantly improved behavioral and synaptic deficits in the present study, tolerability and potential nonspecific adverse effects at the administered dose were not systematically evaluated. Thus, subtle or longer-term effects cannot be fully excluded. Future studies will be required to establish dose-response relationships, determine the minimal effective dose, and define the therapeutic window through comprehensive safety assessments.

## Resource availability

### Lead contact

Further information and requests for resources and code should be directed to the lead contact, Fuhai Ji (jifuhaisuda@163.com), and will be fulfilled by the lead contact.

### Materials availability

This study did not generate new unique reagents.

### Data and code availability

• All data reported in this paper will be shared by the lead contact upon request.
• This study does not report original code.
• Any additional information required to reanalyze the data reported in this paper is available from the lead contact upon request.

## Acknowledgments

This work was supported by the 10.13039/501100001809National Natural Science Foundation of China (grant nos. 82471281, 82072130, 82001126, and 82302465), the Key Medical Research Projects in Jiangsu Province (grant no. ZD2022021), the Key R&D Program Projects in Jiangsu Province (grant no. BE2023709), the Suzhou Key Laboratory of Anesthesiology (SZS2023013), the Suzhou Clinical Medical Center for Anesthesiology (grant no. Szlcyxzxj202102), the Health Talent Plan Project in Suzhou (grant no. GSWS2022007), and the Suzhou Basic Research Pilot Youth Project (grant no. SSD2024065). The funders had no role in the study design, data collection and analysis, decision to publish, or preparation of the manuscript.

## Author contributions

W.-M.Z., Y.-N.G., and Y.-C.W. contributed equally to this work. F.-H.J., H.-Y.L., and X.-W.M. conception and design of research. W.-M.Z., Y.-N.G., Y.-C.W., K.P., and S.-Y.S. performed experiments. W.-M.Z., B.-J.Z., X.-S.S., Y.-F.Y., and L.D. analyzed data. W.-M.Z, K.P., and S.-Y.S. prepared figures. W.-M.Z., K.P., and B.-J.Z. drafted the manuscript. F.-H.J., X.-W.M., H.-Y.L., and H.L. edited and revised the manuscript. All the authors have read and approved the paper.

## Declaration of interests

All authors declare that there are no conflicts of interest.

## STAR★Methods

### Key resources table

**Table:** 

REAGENT or RESOURCE	SOURCE	IDENTIFIER
**Antibodies**		

Rabbit anti-Igfbp2	Abcam	Cat# ab188200; RRID: AB_2938998
Rabbit anti-PSD95	Abcam	Cat# ab238135; RRID: AB_2895158
Rabbit anti- Synapsin-1	Abcam	Cat# ab64581; RRID: AB_1281135
Rabbit anti-GAPDH	GoodHere	Cat# AB-P-R001; RRID: AB_3096355
Goat anti-rabbit IgG	Jackson ImmunoResearch	Cat# 111-035-003; RRID: AB_2313567
Mouse anti-Igfbp2	Santa Cruz	Cat# sc-515134; RRID: AB_3675206
Mouse anti-Igfbp2	R&D Systems	Cat# MAB797; RRID: AB_2264599
Rabbit anti-Sox9	Sigma	Cat# AB5535; RRID: AB_2239761
Rabbit anti-IBA1	Abcam	Cat# ab178847; RRID: AB_2832244
Rabbit anti-NeuN	CST	Cat# 24307; RRID: AB_2651140
Alexa Fluor 488-*anti*-Rabbit secondary antibody	Invitrogen	Cat# A21206; RRID: AB_2535792
Alexa Fluor 555-*anti*-Mouse secondary antibody	Invitrogen	Cat# A31570; RRID: AB_2536180

**Bacterial and virus strains**		

rAAV-hSyn-EGFP-5′miR-30a-shRNA (Igfbp2)-3′miR-30a-WPREs	BrainVTA	N/A
rAAV-hSyn-EGFP-5′miR-30a-shRNA (Scramble)-3′miR-30a-WPREs	BrainVTA	N/A
AAV2/2-Retro-Vglut2-Cre	BrainVTA	Cat# PT-1011

**Chemicals, peptides, and recombinant proteins**		

Recombinant Igfbp2 protein	MCE	Cat# HY-P74846

**Software and algorithms**		

ANY-maze	Stoelting	https://www.any-maze.com
MATLAB 2017b	MathWorks	https://ww2.mathworks.cn/en/products.html?s_tid=gn_ps
GraphPad Prism 10.0	GraphPad Software	https://www.graphpad.com/scientific-software/
ZEN	Zeiss	https://www.zeiss.com/microscopy/us/products/microscope-software/zen.html
ImageJ	National Institutes of Health	https://imagej.nih.gov/ij/index.html
Clampfit	Molecular Devices	https://www.moleculardevices.com/products/axonpatch-clamp-system/acquisition-and-analysissoftware/pclamp-software-suite
MiniAnalysis	Synaptosoft	http://www.synaptosoft.com/MiniAnalysis/

### Experimental model and study participant details

#### Animals

All experiments were performed in adult male mice to reduce potential sex-related variability. C57BL/6J male mice (8 weeks old, 20–25 g) were purchased from Slaccas Laboratory (Shanghai, China). Animals were housed in specific pathogen-free facilities under standard laboratory conditions (23°C–25°C, 40–60% relative humidity, 12-h light/12-h dark cycle) with *ad libitum* access to food and water. Mice were randomly assigned to experimental groups, and sample sizes were determined based on previous behavioral, molecular, and electrophysiological studies conducted in our laboratory.^30^ Investigators were blinded to experimental conditions during both data collection and analysis. All experimental procedures were approved by the Ethics Committee of Soochow University (Suzhou, Jiangsu, China; approval No.: 202408A0217) and conducted in strict accordance with the ARRIVE guidelines.

### Method details

#### Behavioral tests

Mice were acclimated to the testing room for 1 h prior to each behavioral assay. All behavioral tests were performed and analyzed in a blinded manner, with experimenters unaware of group assignments throughout testing and data analysis.

##### Novel object recognition test

The novel object recognition test was performed following procedures described in previous study.^31^ An open-topped arena (40 × 40 × 30 cm) was placed in a dimly lit room. During the training phase, mice were placed in the arena and allowed to freely explore two identical objects for 10 min. After a 90-min inter-trial interval, one the familiar object was replaced with a novel object during the test phase. The time spent exploring the novel object and the number of novel object investigations were recorded over a 10-min period using the ANY-maze behavioral tracking system (Stoelting Co., USA).

##### Y-maze test

The Y-maze test was conducted as previously described.^31^ The apparatus consisted of three arms arranged at 120° angles (each arm: 30 × 4.5 cm, wall height: 15 cm). Distinct visual cues were affixed to the walls at the distal end of each arm. The three arms were designated as the start arm, familiar arm, and novel arm. During the training phase, the novel arm was blocked, and mice freely explored the start and familiar arms for 5 min. Following a 90-min inter-trial interval, the novel arm was opened during the test phase, allowing access to all three arms. The number of entries into and time spent in the novel arm were recorded using the ANY-maze system.

##### Open field test

The open field test was performed as previously described.^32^ Mice were gently placed in a corner of an open-field chamber (40 × 40 × 30 cm), and locomotor activity was recorded for 10 min using an overhead camera coupled with the ANY-maze tracking system. Total distance traveled and time spent in the central zone (20 × 20 cm) were quantified.

##### Elevated plus maze test

The elevated plus maze test was conducted as previously described.^32^ The apparatus consisted of two open arms (30 × 6) and two closed arms (30 × 6 cm, wall height: 15 cm), elevated 60 cm above the floor. Each mouse was placed at the center of the maze, facing an open arm, and allowed to explore freely for 5 min. Time spent in the open arms was recorded and analyzed using the ANY-maze system.

##### Forced swim test

The forced swim test was performed as previously described.^33^ Mice were individually placed in a transparent glass cylinder (height: 70 cm; diameter: 30 cm) filled with water to a depth of 30 cm, maintained at 23 ± 1°C. Sessions were videotaped for 6 min, and immobility time was quantified during the last 5 min.

##### Tail suspension test

The tail suspension test was conducted as previously described.^33^ Each mouse was individually suspended by the tail using adhesive tape positioned 1 cm from the tail tip, at a height of 30 cm above the floor. The test duration was 6 min, and immobility time during the final 5 min was quantified. Behavior was video-recorded throughout the entire session.

#### Stereotaxic surgery

Stereotaxic procedures were performed as previously described.^34^ Mice were anesthetized with 1% sodium pentobarbital (100 mg/kg, intraperitoneally) and placed in a stereotaxic frame (RWD Life Science Inc., Shenzhen, China). rAAV-hSyn-EGFP-5′miR-30a-shRNA (Igfbp2)-3′miR-30a-WPREs (AAV2/9, 5.00E+13 vg mL^−1^, 150 nL; BrainVTA Co. Ltd., Wuhan, China) or rAAV-hSyn-EGFP-5′miR-30a-shRNA (Scramble)-3′miR-30a-WPREs (AAV2/9, 5.00E+13 vg mL^−1^, 150 nL) were bilaterally microinjected into the PFC (coordinates relative to bregma: anterior-posterior, +2.20 mm; medial-lateral, ±0.20 mm; dorsal-ventral, +2.35 mm) at a flow rate of 20 nL/min using a microinjection pump. Following injection, needles remained in place for 10 min before being slowly withdrawn to minimize viral reflux. For cannula implantation experiments, guide cannulas (internal diameter: 0.25 mm; RWD) were bilaterally implanted into the PFC at identical stereotaxic coordinates immediately after viral injection. Dummy cannulas (RWD), secured with dust caps were inserted into the guide cannulas to maintain patency during the recovery period.

#### Drug administration

Recombinant Igfbp2 protein (MCE; Cat# HY-P74846) was dissolved in sterile saline at 10 μg/μL. Igfbp2 was bilaterally infused into the PFC (0.5 μL per side; 5.0 μg, 152 pmol per side) 24 h prior to behavioral testing in mice with neuron-specific Igfbp2 deficiency.^35^ An equal volume of sterile saline was bilaterally infused into the PFC as a vehicle control. Heat-denatured Igfbp2 was infused using the same volume and infusion parameters as an additional protein control. Microinjectors extending 0.5 mm beyond the guide cannula tips were inserted and left in place for 1 min to stabilize. Either sterile saline, Igfbp2 solution, or heat-denatured Igfbp2 was then infused over 1 min. Microinjectors were kept in place for an additional 1 min to allow diffusion before being replaced with obturators.

#### Western blotting

Western blot analysis was performed according to established protocols.^36^ PFC tissues were rapidly dissected and homogenized in ice-cold Radio-Immunoprecipitation Assay (RIPA) lysis buffer supplemented with protease inhibitor cocktail. Protein concentrations were quantified using the bicinchoninic acid (BCA) assay. Equal amounts of protein were separated on 10–15% SDS-PAGE gels and electrotransferred onto polyvinylidene fluoride (PVDF) membranes. Membranes were blocked with 5% non-fat milk in Tris-buffered saline containing 0.1% Tween 20 (TBST) for 2 h at room temperature, then incubated with primary antibodies overnight at 4 °C. Following three 15-min washes with TBST, membranes were incubated with horseradish peroxidase (HRP)-conjugated secondary antibodies for 2 h at room temperature. Immunoreactive bands were visualized using enhanced chemiluminescence (ECL) reagent (Thermo Fisher Scientific) and quantified by densitometry using ImageJ software (NIH).

Primary antibodies included rabbit anti-Igfbp2 (1:500, ab188200, Abcam), rabbit anti-PSD95 (1:500, ab238135, Abcam), rabbit anti-Synapsin-1 (1:500, ab64581, Abcam), and rabbit anti-GAPDH (1:1000, AB-P-R001, GoodHere). The secondary antibody was goat anti-rabbit IgG (1:50,000, 111-035-003, Jackson ImmunoResearch). Protein expression levels were normalized to GAPDH as an internal loading control.

#### Whole-cell electrophysiological recording

Electrophysiological recordings were performed as previously described.^37^ Mice were deeply anesthetized with 1% sodium pentobarbital (100 mg/kg, intraperitoneally) and transcardially perfused with 20 mL of ice-cold, oxygenated (95% O_2_ and 5% CO_2_) slice-cutting solution containing (in mM): 93 N-methyl-D-glucamine, 93 HCl, 2.5 KCl, 1.25 NaH_2_PO_4_, 10 MgSO_4_, 30 NaHCO_3_, 25 glucose, 20 HEPES, 5 sodium ascorbate, 3 sodium pyruvate, and 2 thiourea (pH 7.3–7.4, 300–310 mOsm). Following decapitation, brains were rapidly extracted and 300-μm -thick coronal sections containing the PFC were prepared using a vibrating microtome (VT1200S; Leica, Germany). Brain slices were initially incubated in oxygenated artificial cerebrospinal fluid (aCSF) at 32 °C for at least 30 min, then gradually equilibrated to room temperature (24°C–26°C) before recording. The aCSF contained (in mM): 126 NaCl, 2.5 KCl, 1.25 NaH_2_PO_4_, 2 MgSO_4_, 10 glucose, 26 NaHCO_3_, and 2 CaCl_2_ (pH 7.3–7.4, 300–310 mOsm).

For recordings, individual slices were transferred to a submerged recording chamber continuously perfused with oxygenated aCSF (1–2 mL/min) at room temperature. Neurons in the PFC were visualized using infrared differential interference contrast (IR-DIC) video microscopy with a 40× water-immersion objective (BX51WI, Olympus, Japan). Patch pipettes with resistances of 3–5 MΩ were fabricated from borosilicate glass capillaries using a horizontal pipette puller (P-1000, Sutter Instrument, USA). The internal pipette solution contained (in mM): 133 potassium gluconate, 18 NaCl, 0.6 EGTA, 10 HEPES, 2 Mg-ATP, and 0.3 Na_3_-GTP (pH 7.2 adjusted with KOH, 280–300 mOsm). Whole-cell recordings were acquired using a MultiClamp 700B amplifier (Molecular Devices, USA) controlled by Clampex 10.6 software. Signals were low-pass filtered at 2 kHz and digitized at 10 kHz using a Digidata 1440A analog-to-digital converter (Axon Instruments, USA). Recordings were excluded if series resistance exceeded 20 MΩ or changed by more than 20% during recording.

For current-clamp recordings, action potentials (APs) were evoked by injecting depolarizing current pulses of increasing amplitude (500 ms duration, 40–320 pA, in 40-pA increments). For voltage-clamp recordings, neurons were held at −70 mV, and spontaneous excitatory and inhibitory postsynaptic currents (sEPSC and sIPSC) were continuously recorded for 4 min.

#### Immunofluorescence staining

Mice were transcardially perfused with ice-cold 0.9% saline followed by 4% paraformaldehyde in 0.1 M phosphate buffer (pH 7.4). Brain sections were post-fixed overnight in 4% paraformaldehyde at 4°C, cryoprotected in 30% sucrose solution, and sectioned coronally at 30 μm thickness using a cryostat microtome. Free-floating sections were washed three times in phosphate-buffered saline (PBS; 10 min each), blocked in PBS containing 5% normal donkey serum and 0.3% Triton X-100 for 1 h at room temperature, then incubated with primary antibodies diluted in blocking solution overnight at 4 °C. Following three PBS washes (10 min each), sections were incubated with fluorophore-conjugated secondary antibodies diluted in PBS for 1 h at room temperature in the dark. After three additional PBS washes (10 min each), sections were counterstained with 4′,6-diamidino-2-phenylindole (DAPI; 1:5000) for nuclear visualization, mounted on glass slides, and coverslipped with anti-fade mounting medium.

Primary antibodies included mouse anti-Igfbp2 (1:200, sc-51534, Santa Cruz Biotechnology), anti-Igfbp2 (1:500, MAB797, R&D System), rabbit anti-Sox9 (1:500, AB5535, Sigma), rabbit anti-IBA1 (1:200, ab178847, Abcam), and rabbit anti-NeuN (1:200, 24307, Cell Signaling Technology). Secondary antibodies were donkey anti-rabbit Alexa Fluor 488 (1:500, Invitrogen) and donkey anti-mouse Alexa Fluor 555 (1:500, Invitrogen). Fluorescence images were acquired using a confocal laser scanning microscope (Zeiss, LSM900) with appropriate excitation and emission filters.

#### Golgi staining

Golgi staining was performed using the FD Rapid Golgi Staining Kit (PK401, FD NeuroTechnologies, USA) according to the manufacturer’s instructions. Briefly, freshly dissected brain samples were immersed in a 1:1 mixture of solutions A and B and incubated in the dark at room temperature for 14 days with solution changes every 3–4 days. Samples were subsequently transferred to solution C and incubated in the dark at room temperature for an additional 5 days. Brain tissues were then embedded in optimal cutting temperature (OCT) compound and rapidly frozen at −80 °C for 24 h. Coronal sections (100 μm) were cut using a cryostat microtome (Leica, Wetzlar, Germany), and mounted on gelatin-coated slides, and processed according to the kit protocol. Stained sections were dehydrated through graded ethanol series, cleared in xylene, and coverslipped with Permount mounting medium.

Dendritic spine analysis was performed on layer II/III pyramidal neurons in the PFC. For each neuron, three secondary or tertiary basal dendrite segments (minimum 30 μm in length, located >50 μm from the soma) were randomly selected for analysis. High-magnification images were acquired using a confocal microscope equipped with a 63× oil-immersion objective lens (Zeiss, LSM900). Dendritic spine density (number of spines per 10 μm dendritic length) was quantified using ImageJ software (NIH) by an experimenter blinded to experimental conditions.

### Quantification and statistical analysis

Animals with misplaced viral injections or cannula implantations, as verified by post-hoc histological analysis, were excluded from statistical analysis. All data are presented as mean ± standard error of the mean (SEM). Normality of data distribution was assessed using the Shapiro-Wilk test, and homogeneity of variance was evaluated using Bartlett’s test. For comparisons between two groups, unpaired two-tailed Student’s *t* test were performed. For comparisons involving multiple groups or repeated measures, two-way repeated-measures analysis of variance (ANOVA) followed by Sidak’s post-hoc multiple comparisons test was used. All statistical analyses were conducted using GraphPad Prism 10.0 (GraphPad Software). A probability value of *p* < 0.05 was considered statistically significant.
