# Supplementary material for: Neuronal Igfbp2 deficiency in the prefrontal cortex impairs cognition through synaptic dysfunction in male mice
Source: iScience. 2026 Apr 6;29(5):115629. doi: 10.1016/j.isci.2026.115629 (PMC13122697; doi:10.1016/j.isci.2026.115629)
Supplement: Document S1. Figures S1–S7 [file mmc1.pdf]

iScience, Volume *29*

Supplemental information

Neuronal Igfbp2 deficiency in the prefrontal cortex impairs cognition through synaptic dysfunction in male mice

Weiming Zhao, Yanan Gao, Yichan Wang, Ke Peng, Shaoyong Song, Yufan Yang, Baojian Zhao, Xisheng Shan, Li Deng, Ruixia Weng, Hong Liu, Xiaowen Meng, Huayue Liu, and Fuhai Ji

Supplemental information

Document S1. Figures S1-S7.

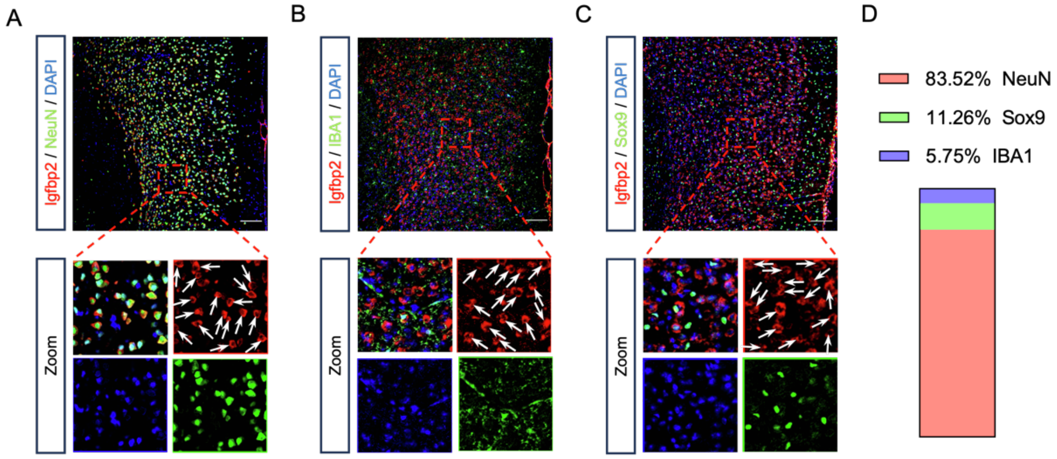

Figure S1. Validation of Igfbp2 immunostaining using a second independent antibody. Related to Figure 1.

(A) Representative immunofluorescence image of Igfbp2 (red) and NeuN (green) in the PFC.

(B) Representative immunofluorescence image of Igfbp2 (red) and IBA1 (green) in the PFC.

(C) Representative immunofluorescence image of Igfbp2 (red) and Sox9 (green) in the PFC.

(D) Quantitative analysis of Igfbp2 colocalization with different cell types in the PFC. Scale bar: 100 μm; n = 4 brain sections from 4 mice per group.

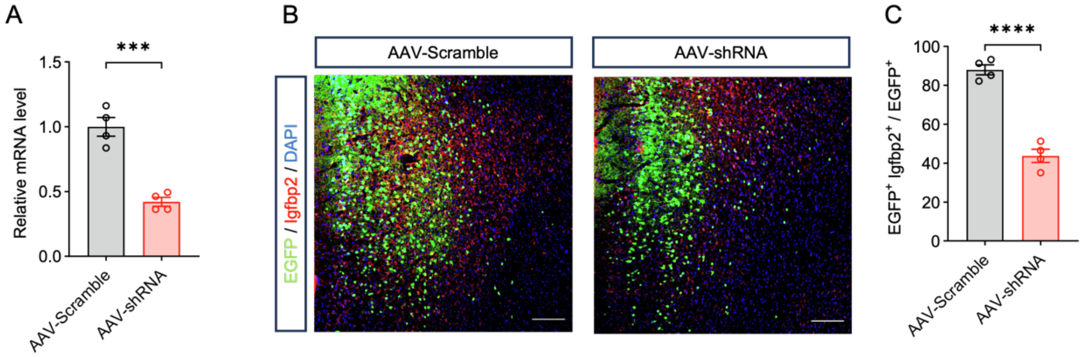

Figure S2. Validation of Igfbp2 knockdown efficiency in the PFC. Related to Figure 2.

(A) qRT-PCR analysis of Igfbp2 mRNA levels in PFC tissue, n = 4 mice per group.

(B) Representative immunofluorescence image of Igfbp2 (red) and EGFP (green) in the PFC; scale bar: 100 μm; n = 4 brain sections from 4 mice per group.

(C) Quantitative analysis of Igfbp2 colocalization with EGFP in the PFC.

Analyzed by unpaired *t* test; ***$P < 0.001$; ****$P < 0.0001$. Data are presented as means ± SEM.

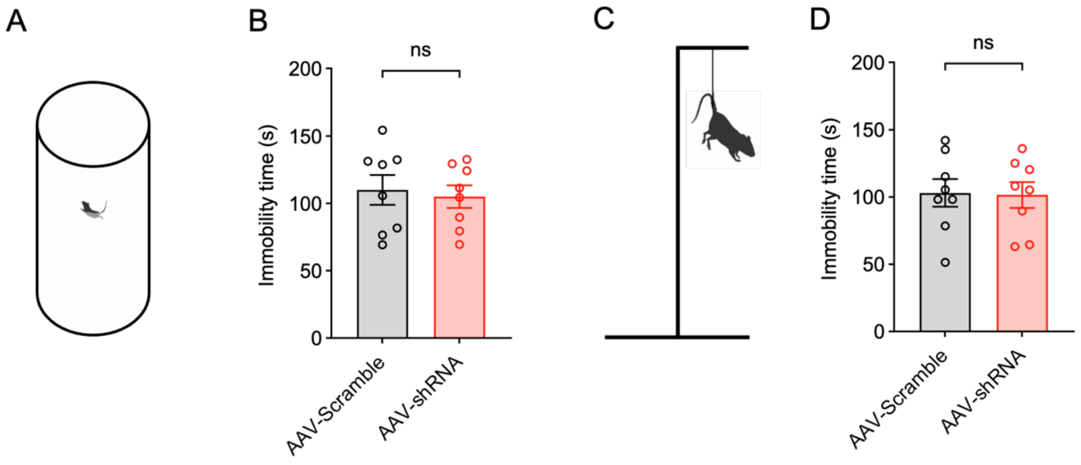

Figure S3. Neuron-specific Igfbp2 deficiency in the PFC does not induce depression-like behavior. Related to Figure 2.

(A) Schematic of the forced swim test.

(B) Immobility time in the forced swim test.

(C) Schematic of the tail suspension test.

(D) Immobility time in the tail suspension test.

Analyzed by unpaired *t* test; n = 8 mice per group; ns: not significance. Data are presented as means ± SEM.

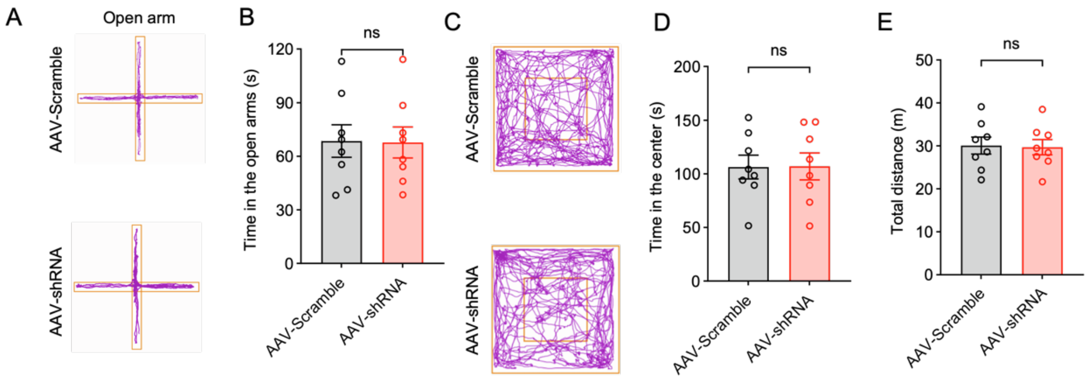

Figure S4. Neuron-specific Igfbp2 deficiency in the PFC does not induce anxiety-like behavior or motor deficits. Related to Figure 1.

(A) Representative trajectory plots from the elevated plus maze test.

(B) Time spent in the open arms of the elevated plus maze test.

(C) Representative trajectory plots from the open field test.

(D) Time spent in the center region of the open field arena.

(E) Total distance traveled in the open field arena.

Analyzed by unpaired *t* test; n = 8 mice per group; ns: not significance. Data are presented as means ± SEM.

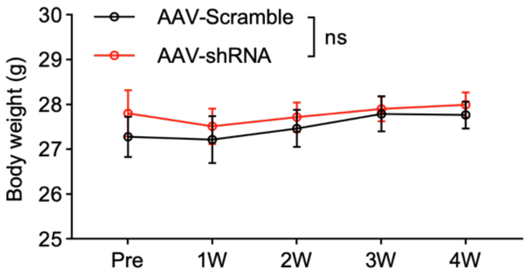

Figure S5. Neuron-specific Igfbp2 deficiency in the PFC does not affect body weight. Related to Figure 2.

Quantification of body weight showing no significant difference between the AAV-Scramble and AAV-shRNA groups.

Analyzed by two-way repeated-measure ANOVA; n = 8 mice per group; ns: not significance. Data are presented as means ± SEM.

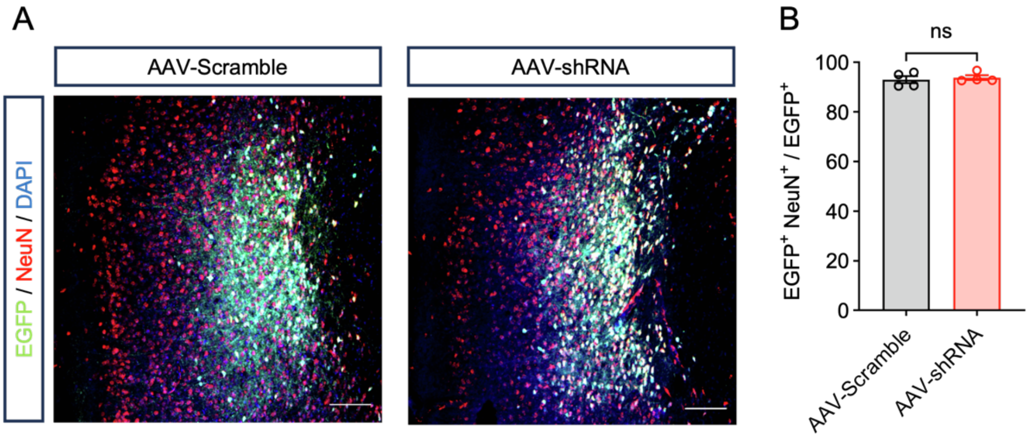

Figure S6. Neuron-specific Igfbp2 deficiency in the PFC does not affect the number of neurons. Related to Figure 3.

(A) Representative confocal images of NeuN-positive cells in PFC, scale bar: 100 μm.

(B) Quantitative analysis of NeuN-positive cells in PFC.

Analyzed by unpaired *t* test; n = 4 brain sections from 4 mice per group; ns: not significance. Data are presented as means ± SEM.

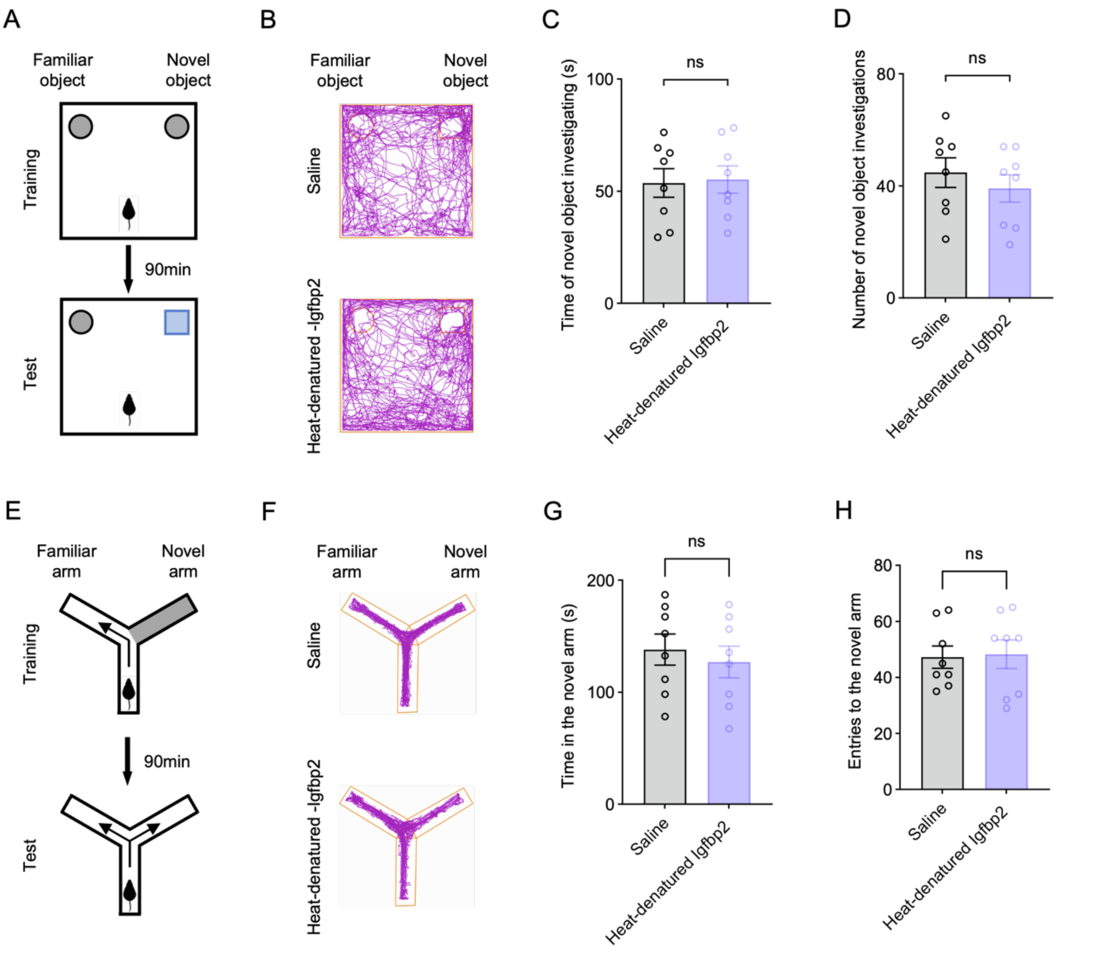

Figure S7. Exogenous supplementation with heat-denatured Igfbp2 in the PFC fails to rescue the cognitive deficits induced by neuron-specific Igfbp2 deficiency. Related to Figure 5.

(A) Schematic of the novel object recognition test, depicting the training phase (top) and the test phase (bottom).

(B) Representative trajectory plots from the novel object recognition test.

(C) Quantification of time spent investigating the novel object during the test phase.

(D) Quantification of the number of investigations of the novel object during the test phase.

(E) Schematic of the Y-maze test, depicting the training phase (top) and the test phase (bottom).

(F) Representative trajectory plots from the Y-maze test.

(G) Quantification of time spent in the novel arm during the test phase.

(H) Quantification of the number of entries into the novel arm during the test phase.

Analyzed by unpaired *t* test; n = 8 mice per group; $^{**}P < 0.01$. Data are presented as means ± SEM.
